# Infectious disease outbreak prediction using media articles with machine learning models

**DOI:** 10.1038/s41598-021-83926-2

**Published:** 2021-02-24

**Authors:** Juhyeon Kim, Insung Ahn

**Affiliations:** 1 Department of Data-Centric Problem Solving Research, Korea Institute of Science and Technology Information, Yuseong-gu, Daejeon, Korea; 2 Center for Convergent Research of Emerging Virus Infection, Korea Research Institute of Chemical Technology, Yuseong-gu, Daejeon, Korea

**Keywords:** Infectious diseases, Epidemiology, Computer science, Information technology

## Abstract

When a newly emerging infectious disease breaks out in a country, it brings critical damage to both human health conditions and the national economy. For this reason, apprehending which disease will newly emerge, and preparing countermeasures for that disease, are required. Many different types of infectious diseases are emerging and threatening global human health conditions. For this reason, the detection of emerging infectious disease pattern is critical. However, as the epidemic spread of infectious disease occurs sporadically and rapidly, it is not easy to predict whether an infectious disease will emerge or not. Furthermore, accumulating data related to a specific infectious disease is not easy. For these reasons, finding useful data and building a prediction model with these data is required. The Internet press releases numerous articles every day that rapidly reflect currently pending issues. Thus, in this research, we accumulated Internet articles from Medisys that were related to infectious disease, to see if news data could be used to predict infectious disease outbreak. Articles related to infectious disease from January to December 2019 were collected. In this study, we evaluated if newly emerging infectious diseases could be detected using the news article data. Support Vector Machine (SVM), Semi-supervised Learning (SSL), and Deep Neural Network (DNN) were used for prediction to examine the use of information embedded in the web articles: and to detect the pattern of emerging infectious disease.

## Introduction

The spread of middle East respiratory syndrome (MERS) in 2015 caused 185 confirmed cases and 36 deaths^1^. The first outbreak of MERS in the Republic of Korea (Korea) occurred on May 2015, after a 68-year-old man returned from a business trip to several Middle East countries. As Korea could not predict if MERS might flow across the border, MERS not only threatened public health, but also caused huge economic loss in many different categories, including the tourist industry and social activity. Such a situation indicates that judging if an infectious disease will influx from other countries or not in advance is an important issue to minimize the damage that ensues. MERS was first reported in September 2012 from Saudi Arabia, and was reported from several European countries, before MERS occurred in Korea during 2015^1^. As MERS was not a commonly known disease in Korea, there was indifference to it before it occurred. However, if it was possible to predict that MERS might flow into Korea while it was spreading around the world, Korea could have prepared for the outbreak of the MERS to minimize the damage it caused. On the other hand, while MERS was spreading through several continents, Ebola spread through 5 different countries in Western Africa, infecting more than 6,500 people, and killing more than 3000 people^2^. Even though Ebola outbreaks occurred a few times on the Africa continent, the 2014 pandemic was the biggest one^3^. The 2014 Ebola pandemic in Western Africa showed a fatality rate of over 50%. However unlike MERS, Ebola, did not spread throughout other continents.

Many different infectious diseases threaten lives worldwide. Some diseases, like MERS, cause pandemics, spreading from country to country over continents, while some do not spread over continents, but like Ebola, circulate only in a few countries. As infectious disease issues arise worldwide, many researches were conducted to estimate and predict the occurrence of infectious diseases. Authors of^4,5^ developed infectious disease spread simulation models using mathematical models. These research efforts utilized susceptible infected recovered (SIR) models to build an infectious disease spread simulation model, and suggest strategies to control infectious disease and maximize the effect of vaccination with the results from the simulation models. Commonly, these SIR simulation models concern the population of the area the model is based on and the characteristic of the disease, such as infection rate, incubation rate, and recovery rate. Some research considers the passengers of flights crossing borders to explain how infectious disease spreads abroad^6^. Moreover, authors of^7^ claimed that infectious disease epidemics can be related to climate and climatic events, such as El Nino. According to the existing research reports above, the occurrence of infectious diseases varies depending on many different reasons, such as climate, lifestyle of countries, diplomatic relations between countries, or population. Thus, it is important to collect and use the latest data for future infectious disease outbreak prediction. However, the degree of these features for each country varies according to the passage of time. For example, El Nino changes the climatic attributes throughout the world, digitalization changes the lifestyle of human, and the number of travelers or the amount of trade between countries may change dramatically for political reasons. Consequently, constructing an infectious disease outbreak prediction model considering all these features is a challenging matter. However, as infectious disease spreads based on all these features, it may be possible to assert that the rate of particular infectious disease occurrence in a particular country connotes the information mentioned above. This means that we can assume that some infectious disease occurs in a particular country, because the conditions of certain features, such as climate, population, lifestyle, and the number of incoming travelers exceed thresholds for the disease in that country. With this assumption, it is possible to forecast if an infectious disease that has not occurred recently in a particular country will break out or not in that country, by analyzing the patterns of many different types of infectious diseases occurring in different countries.

Normally, when an infectious disease breaks out, the press media publish articles concerning the disease. When the seriousness of the disease becomes higher for some reason, like the increase in the number of infected people, the number of published articles also increases. In other words, the number of articles reported related to a particular disease in a particular country reflects how severe the disease is in that country. Furthermore, media articles and reports are updated in real-time through the Internet service worldwide, which offers the advantage of accumulating the latest data immediately, while collecting actual surveillance data of numerous disease types from countries worldwide is a difficult task^8^. Therefore, various attempts have been made to utilize media article data to predict an epidemic outbreak. Most of these studies, utilizing data from online media articles, try to figure out the epidemics occurring in specific country. In study^9^, media articles related to specific infectious diseases that occurred in the United States, China, and India respectively were collected, and based on this, the temporal topic trend was compared with the actual disease case count. The outbreak of whooping cough, rabies, salmonellosis, and E. coli infection in the United States, H7N9, hand, foot, and mouth disease, and dengue in China, and acute diarrheal disease, dengue, and malaria in India were estimated by proposing method. This allowed the authors to successfully capture the dynamics of disease outbreak by the temporal topic trends obtained through media articles. In other words, the degree of the temporal topic trend for a specific infectious disease in such a specific country can actually indicate the severity of the infectious disease in that country. Furthermore, in a study proposing a method to monitor infectious diseases using online news media data, the proposed model was applied to the outbreak of dengue fever in India and the outbreak of zika virus in Brazil^10^. In the study, using the collected international newspaper data and local newspaper data, the number of news reports related to each disease was calculated, and how similar the number of actual disease cases was. The authors of the study argue that there is a possibility to build a surveillance system using news data even in developing countries that do not have a surveillance systems yet. The authors of^8,11^ suggested a method of predicting the occurrence of infectious diseases by extracting keywords with high relevance to specific infectious diseases instead of the simple number of occurrences of media articles related to a specific infectious disease. All of the aforementioned studies suggest a method to estimate the number of patients with a specific infectious disease in a specific country using online media article data. The existing studies showed the potential that online media article data can make a great contribution to the prediction of infectious diseases. However, since previous studies have focused on establishing an outbreak surveillance system, such as measuring the number of existing infectious diseases in a specific region, there is a limitation that only a limited number of infectious diseases can be treated in a limited number of countries. In other words, it cannot handle various kinds of infectious diseases occurring in various countries because the country and the type of infectious disease are specified. In addition, the previous infectious disease prediction studies have successfully established a surveillance system for infectious diseases that have seasonality or have been present in certain countries, but there is a limitation that it is impossible to predict the occurrence of infectious diseases that have not occurred. Thus, this study proposes a methodology for predicting the occurrence of various infectious diseases that did not occur for 6 months in various countries around the world by analyzing media article data. The remainder of this paper is organized as follows. “Methods” section explains which data is used for infectious disease outbreak prediction, and introduces the three machine learning models, semi-supervised learning (SSL), support vector machine (SVM), and deep neural network (DNN). “Experiments” section details the performance measures, and the experimental settings and results. Finally, “Results” and “Conclusion” sections present our discussions and conclusions, respectively.

## Methods

**Figure 1:**
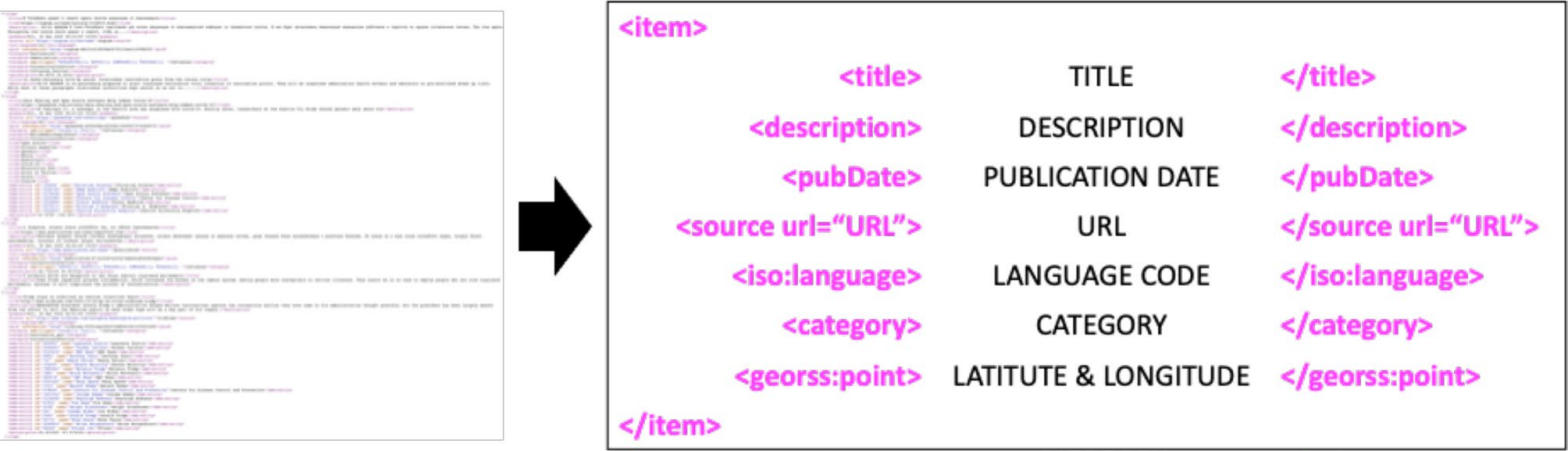
Examples of data provided by Medisys in the form of RSS and components of RSS provided by Medisys.

**Figure 2:**
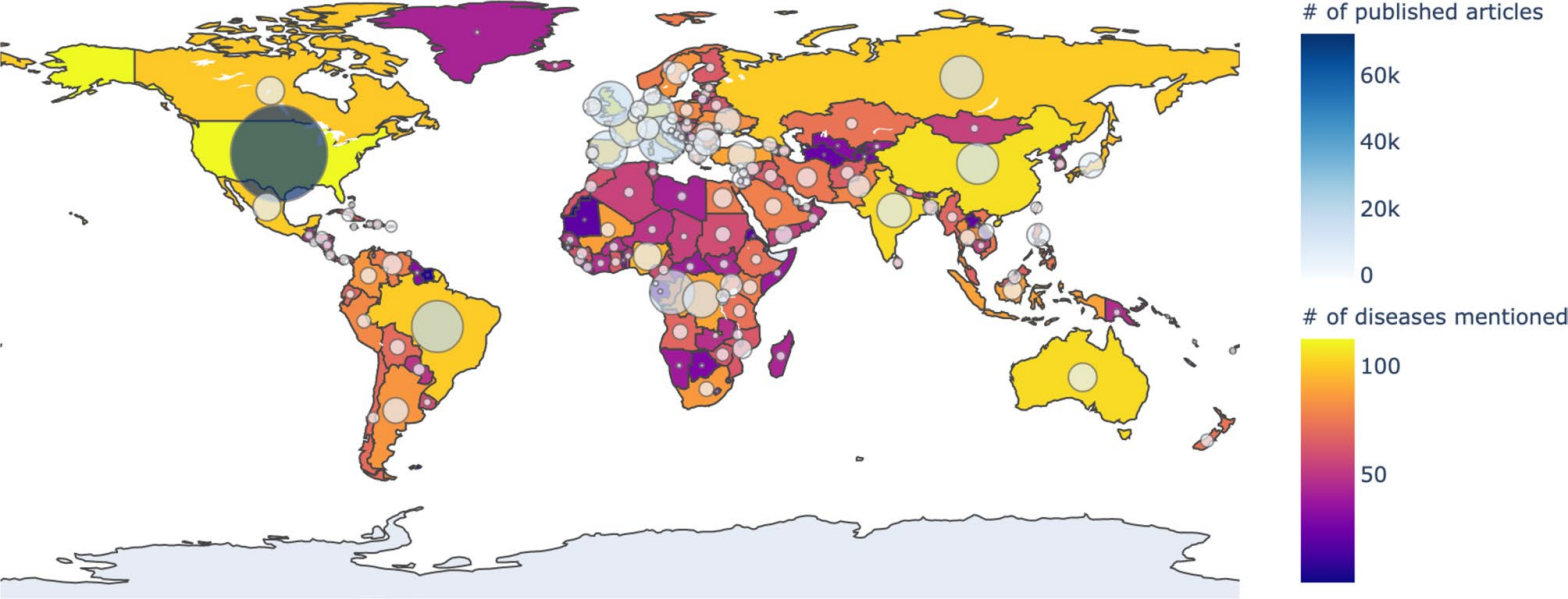
The number of articles published in each country collected from Medisys from January to December 2019: the closer the color of the country to yellow, the more diseases occurred, and the larger the circle, the more articles have occurred. The figure was created in Python3 using the Basemap Toolkit.

**Figure 3:**
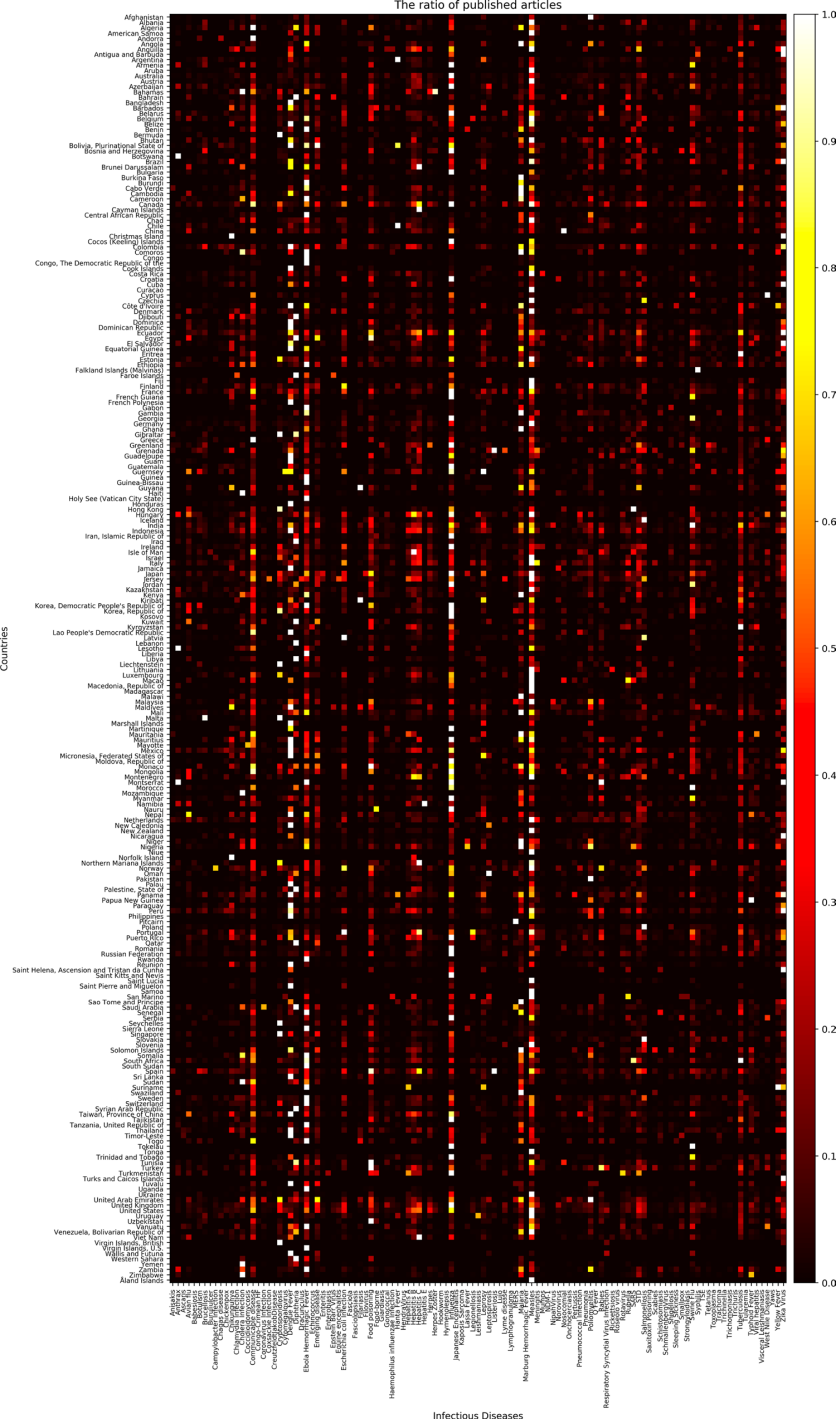
The number of articles related to each disease by country collected from Medisys for 2019 from January to December (data is normalized, thus the brighter the color, the more articles; the darker, the less articles).

Nowadays, as the Internet service is supplied worldwide, people obtain information using the Internet service easily and rapidly. Even news articles are being published through the Internet, unlike in the past, when they were printed on paper and delivered. Accordingly, articles and reports related to infectious diseases are also being published and updated through the Internet media in real-time. In other words, unlike in the past, the Internet media has made it easy to obtain information about the seriousness of infectious disease issues around the world today. Thus, in this research, we collected articles and reports related to 115 different infectious diseases from Medisys, to predict if a particular infectious disease that had not occurred for several months in a particular country will break out in that country. Medisys serves news articles and reports of infectious disease published worldwide every day in real-time^12^. Articles and reports provided by Medisys are classified by disease, and include the date and time they were published, and the latitude and longitude of the information where the outbreak of disease occurred. Every articles are also published in rich site summary (RSS) form. RSS is a method of displaying content primarily used on news or blog sites. If website administrators display website content in RSS format, recipients of this information may use it in different formats. Figure 1 shows examples of data provided by Medisys in RSS form and their components. The information of each article is displayed between < item > and < /item > , and data such as article title, description, publication date, original url, language code, category indicating the name of the disease, latitude and longitude are displayed. Even though the Medisys reports do not provide where the articles are published, it is possible to track where they were published by analyzing the latitude and longitude information. We accumulated data from Medisys for January to December 2019. This data consisted of 115,279 articles published in 237 different countries. As described in Fig. 2, the number of articles per nation, and infectious disease were extracted from the data, and utilized in this study. However, some poor and developing countries, especially if involved in wars, have less opportunity to publish digital data. Furthermore, the population sizes by country also varies which may affect the number of published articles. For these reasons, data is normalized between 0 and 1 by each country to adjust values measured on different scales. Figure 3 shows the reorganized data.

**Figure 4:**
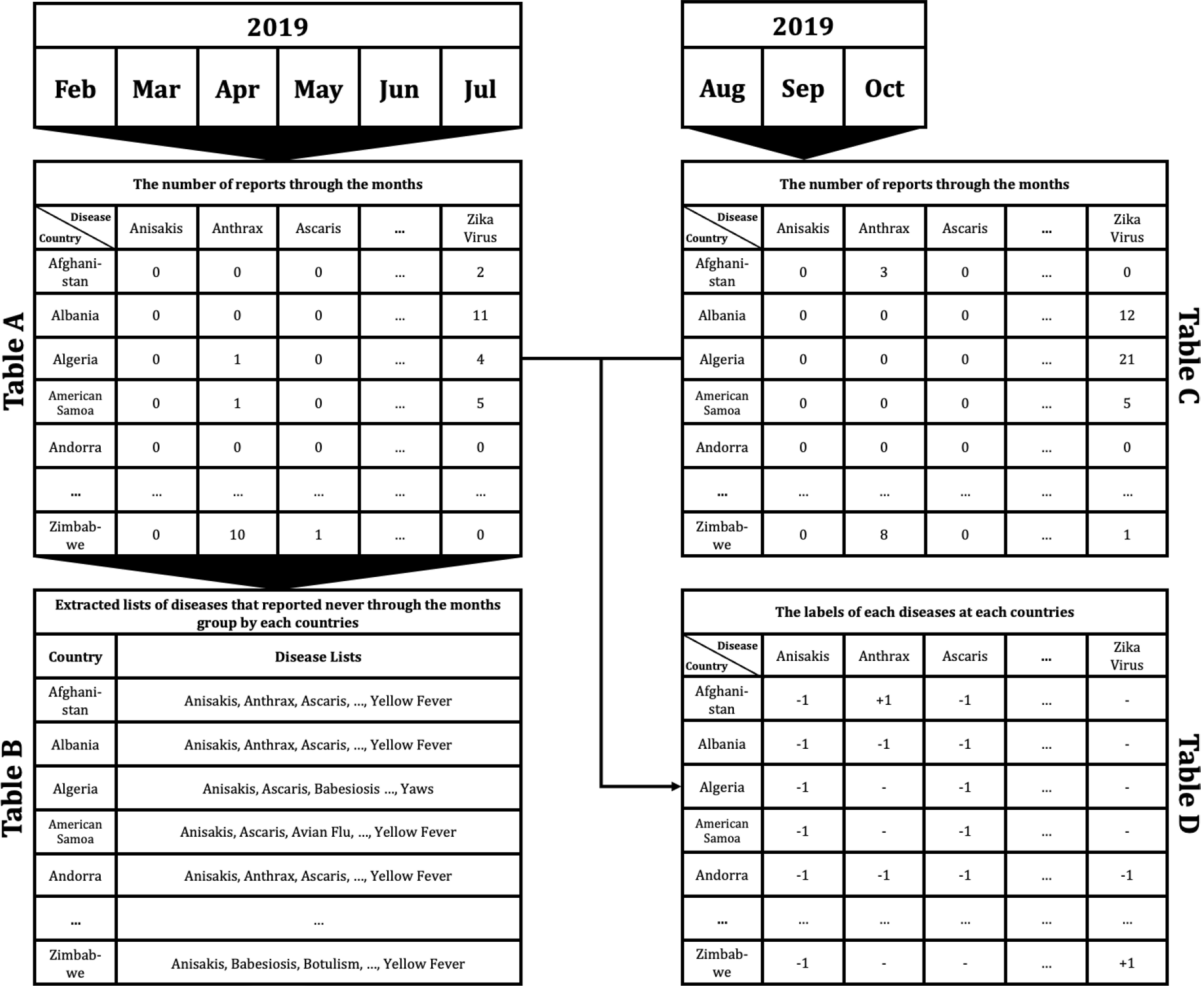
Example of data preprocessing to predict infectious disease outbreak for 3 months after July 2019, using report count data from February to July 2019: Table A indicates the number of reports concerning each disease in each country from February to July 2019. Table B shows the lists of diseases that reported none during the 6 month period February to July 2019 in each country. Table C shows the number of counted reports related to listed diseases in each country from August to October in 2019. Finally, Table D shows the labels of each disease for each country. ‘ + 1′ indicates that the disease occurred in the country between August and October; in contrast, ‘ − 1′ indicates that the disease did not occur, while ‘ − ’ means that the disease had already occurred during the period February to July, thus the disease for the country does not display a label.

To apply the constructed data to machine learning models to predict if disease that had not occurred for several months in a particular country would occur or not, the data set was preprocessed as follows. For example, as shown in Fig. 4, Table A extracted the number of articles related to 115 different diseases by 237 countries during the 6 month period February to July 2019. From Table A, a disease list that contains ‘0′, which means diseases never occurred from each country, was extracted and listed in Table B by country. Each disease listed in Table B is considered, as it may have the potential for outbreak, because it has not yet occurred in each country. Table C is the data from August to October 2019, 3 months after July 2019. Then if the data of a particular disease for a particular country is 0 in both Tables A and C, the label of the disease of the country becomes ‘ − 1′; while when Table A is 0, but Table C is > 0, the label becomes ‘ + 1′. These labels can be arranged as in Table D.

**Figure 5:**
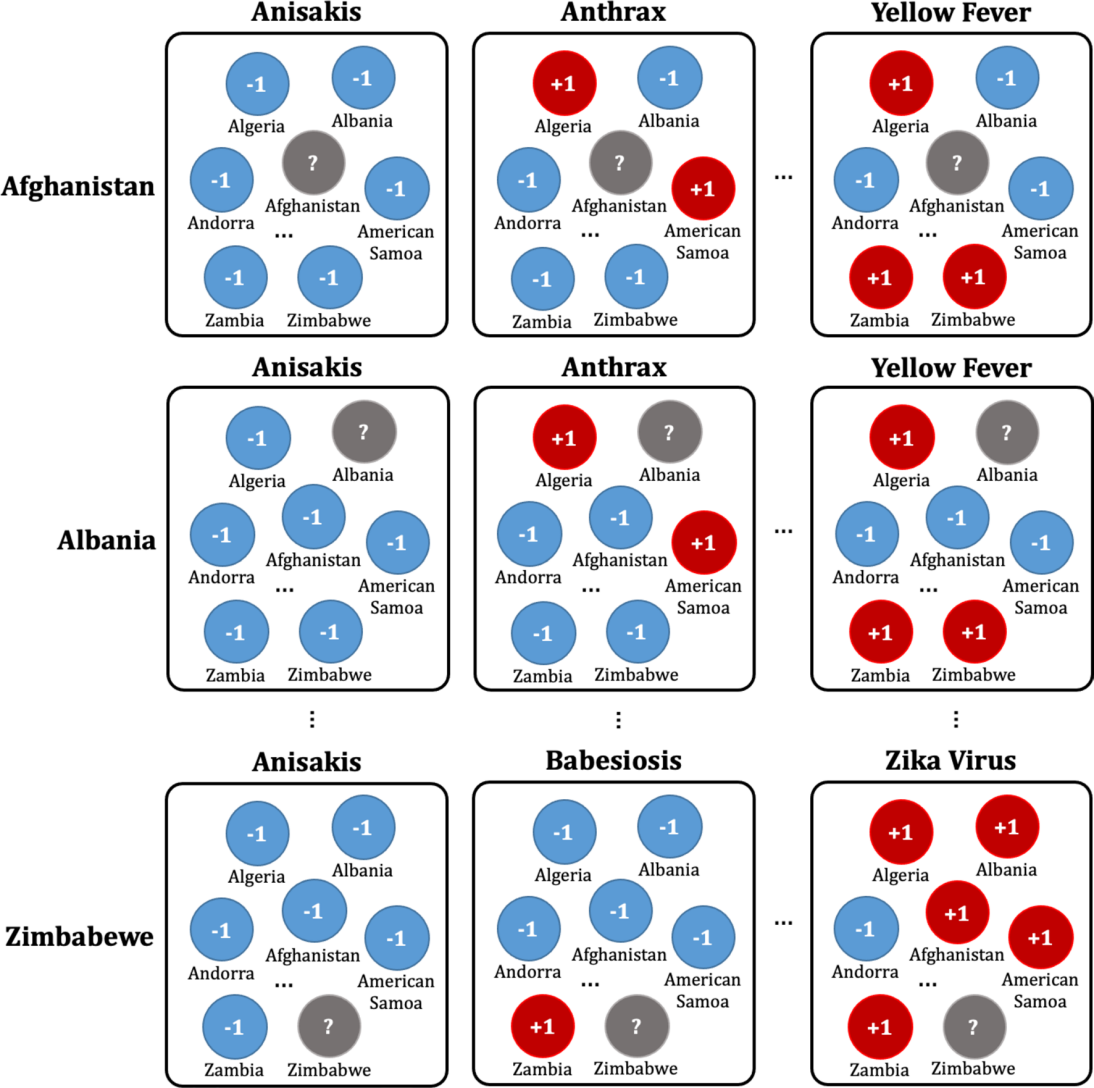
Data set composition for the prediction model for each disease by country: For example, in the first row, the data set of diseases for Afghanistan is listed in the first row of Table B of Fig. 4. Nodes with ‘ + 1′ indicate that the reports related to the disease occurred more than once in the country, while nodes with ‘ − 1′ indicate that the reports related to the diseases never occurred in Table A of Fig. 4.

**Figure 6:**
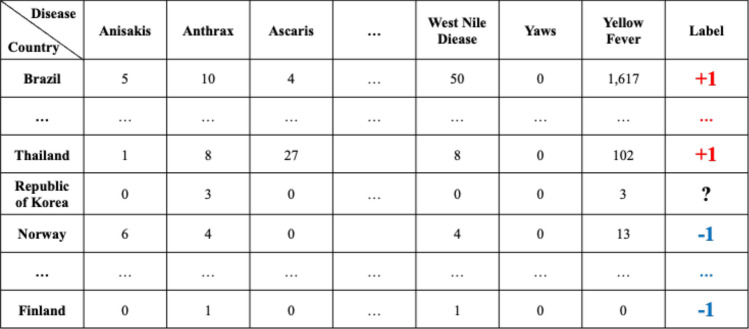
The overall data structure of each square shown in Fig. 5.

Once the data has been preprocessed as shown in Fig. 4, it is possible to select a list of what infectious diseases should be predicted in each country, as shown in Table B, and based on this list, it is possible to create a label set for each country and for each disease, as shown in Table D. With the preprocessed data, the data set for the prediction models of each disease by country can be organized as shown in Fig. 5. Every node in Fig. 5 is composed of data in Table A in Fig. 4. In Fig. 5, if more than a single report related to the infectious disease by country occurred, then ‘ + 1′ is labeled, and in contrast, in the case of a report that was not reported in Table A of Fig. 4, ‘ − 1′ is labeled ,while unlabeled nodes ‘?’ are listed in Table B of Fig. 4. In other words, each square shown in Fig. 5 is a set of labels for predicting disease outbreak in each country, and the overall data structure of each square can be represented as shown in Fig. 6. In Fig. 6, the number in each column is the number of media articles related to each infectious disease in each country that occurred during the specified period. For all unlabeled data, a data set as shown in Fig. 6 is formed based on label of Fig. 5, and each data set is applied to machine learning models to predict the occurrence of a specific infectious disease in a specific country.

**Figure 7:**
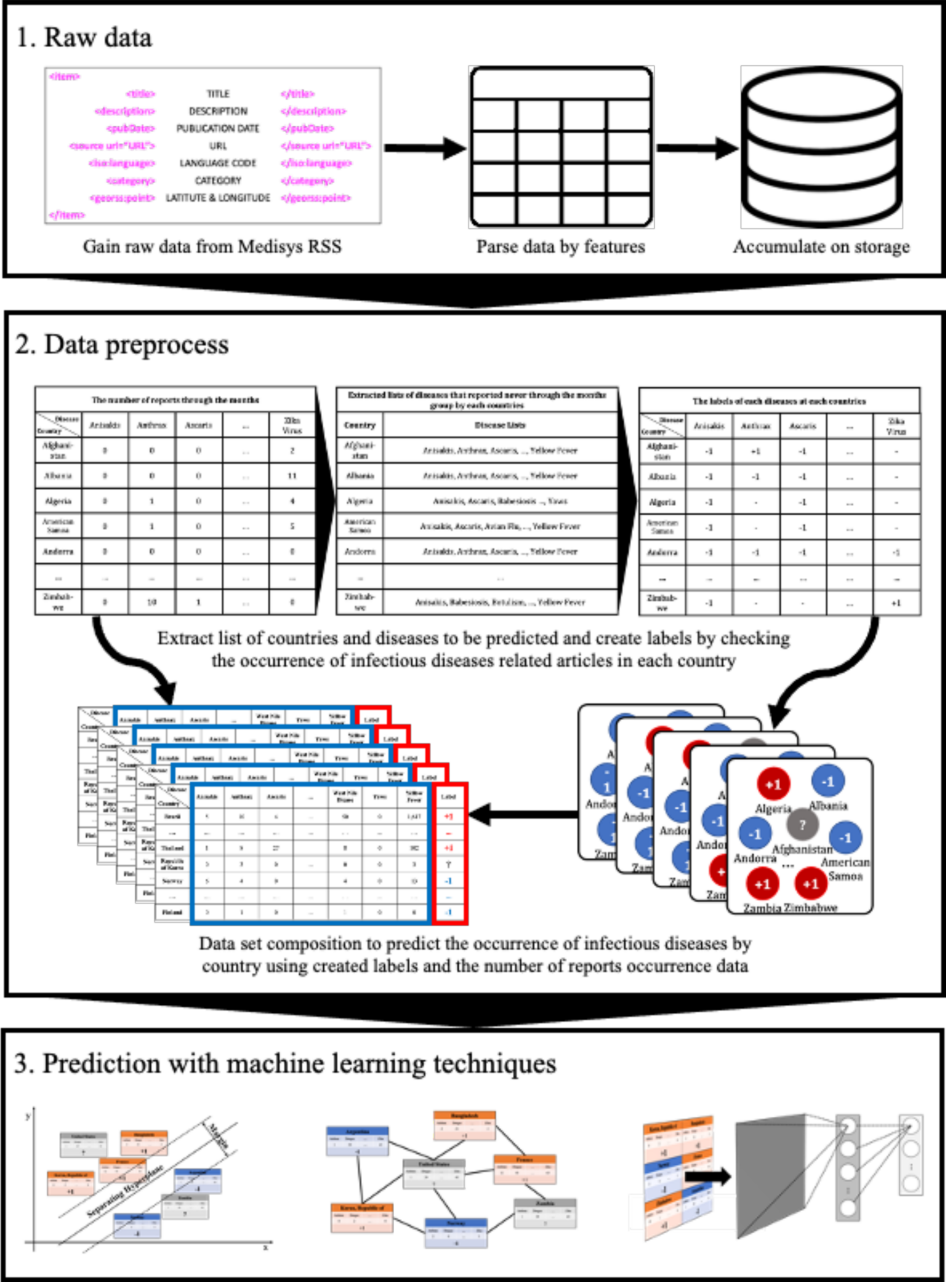
The order of progress from data preprocessing to prediction.

In this research, we adapted three different machine learning models to investigate if early disease outbreak detection would be possible using media articles and reports related to infectious disease, and compared the performance of the models. Three representative models, that is, support vector machine (SVM), which shows good performance consistently through various fields; semi-supervised learning (SSL), which shows good performance when label imbalanced data sets are used; and deep neural network (DNN), which is a trending method showing outstanding performance, were used to perform prediction for disease occurrence. The model parameters of SVM, SSL, and DNN were searched over the following ranges. For SVM, the best prediction performances were identified from the combinations of { γ, C} ∈ {0.0001, 0.001, 0.01, 0.1, 1, 10} × {0.2, 0.4, 0.6, 0.8, 1}^13^. For SSL, k, which is a parameter to decide the number of neighbors was identified from k = {3, 7, 15, 20, 30}, and μ, which is a trade-off parameter, was identified from μ = {0.0001, 0.01, 1, 100, 1000}. Finally, DNN model was organized with 3 layers with batch size of 20 for each step. Dropouts are set as 0.3 for each layer, and Adam gradient descent optimization was applied, while epoch was set as 500. After disease outbreak prediction is made with each model, the model performance is calculated using Table D of Fig. 4 by comparing the prediction results with the corresponding infectious disease 3 months after the last date used as the training data. The order of progress from data preprocessing to prediction can be summarized as shown in Fig. 7.

### Ethics approval and consent to participate

This study did not involve human participants, data, or tissue. Institutional review board approval was not required.

## Experiments

**Figure 8:**
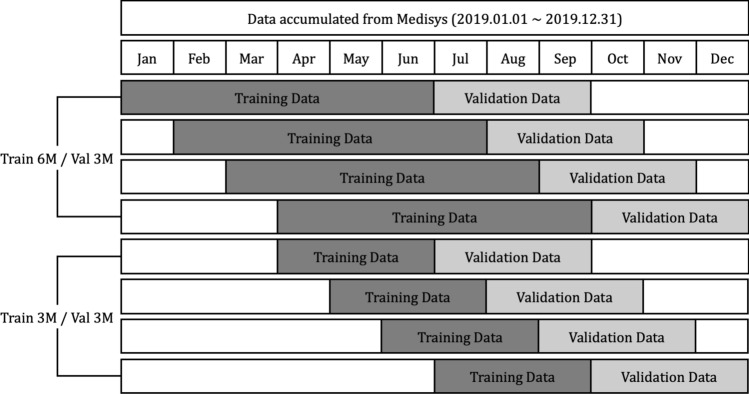
Using data crawled for a year, experiments are set as first, each model being trained using 6 months’ data, and predicting if the disease will outbreak or not; and second, each model being trained using 3 months’ data, and predicting if the disease will outbreak or not.

Media articles and reports that are published from January to December 2019 crawled from Medisys are used in this research. The crawled data includes the title of articles, description, published date and time, disease related to, and the latitude and longitude information. Parsing the data, counts of the number of daily articles related to each disease by country are extracted, and organized as a numerical dataset. A total of 115 different diseases and 237 different countries are concerned with the extracted dataset, and the average count of the number of daily articles is about 1300. Each data point is normalized between 0 and 1. As shown in Fig. 8, experiments are done with two different strategies, setting the length of training data as (6 and 3) months, and the validation data as 3 months, respectively. It is discovered whether each model can predict whether diseases will break out or not by country during the 3 months after the training data of July to September, August to October, September to November, and October to December, respectively.

To measure the performance of each prediction model, AUC, Accuracy, and F1 score are used^14,15^. The AUC assesses the overall value of a classifier, which is a threshold-independent measure of model performance based on the receiver operating characteristic curve that plots the trade-offs between sensitivity and 1—specificity for all possible values of threshold. Accuracy is a measure of the total number of correct predictions when the value of the classification threshold is set to 0. Lastly, the F1 score can be interpreted as the weighted average of the precision and recall, where an F1 score reaches its best value at 1, while the worst score is 0.

## Results

**Figure 9:**
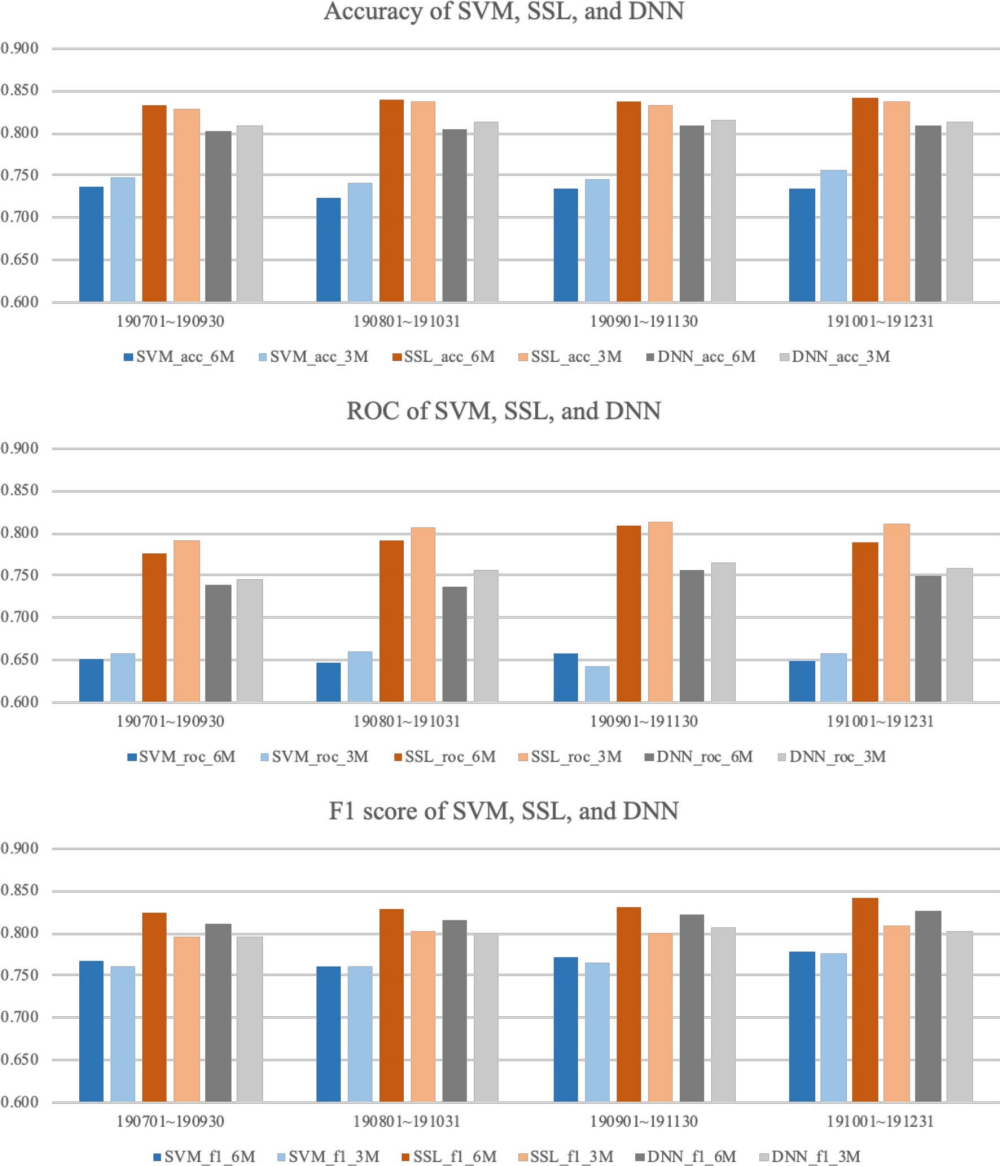
Accuracy, ROC, and F1 score of each validation data set period by each model, respectively.

The results of the experiment are based on the expected accuracy of whether the diseases that had not been reported for (6 or 3) months will break out or not by country. Tables 1 and 2 show a comparison of the results with SVM, SSL, and DNN in terms of the accuracy, ROC, and F1 score. For each of the three models, the best performance was selected by searching over the respective model-parameter space. For each dataset, the best performance among the three models is marked in bold face. In terms of the accuracy, SSL shows the best performance, with an average accuracy of (0.838 and 0.834). In terms of the ROC, SSL delivers outstanding performance, with an average ROC of (0.791 and 0.805). Lastly, even in the F1 score case, SSL produces an average (0.832 and 0.802), which is the best of the three models. Figure 9 summarizes the performance of the three models in bar graphs. Even though SSL shows outstanding performance compared to other two models, SVM and DNN also show reasonable performance, showing average accuracy over 0.7, and F1 score over 0.75.

**Table 1:** Performance comparison using SVM, SSL, and DNN with training set using 6 months of data.

Validation set period	Accuracy			ROC			F1 Score		
	SVM	SSL	DNN	SVM	SSL	DNN	SVM	SSL	DNN
2019.07.01–2019.09.30	0.736	**0.834**	0.803	0.651	**0.775**	0.739	0.768	**0.825**	0.811
2019.08.01–2019.10.31	0.724	**0.839**	0.805	0.646	**0.791**	0.737	0.760	**0.829**	0.816
2019.09.01–2019.11.30	0.735	**0.837**	0.809	0.657	**0.810**	0.756	0.771	**0.831**	0.822
2019.10.01–2019.12.31	0.734	**0.842**	0.809	0.648	**0.788**	0.751	0.778	**0.842**	0.826
Average	0.732	**0.838**	0.806	0.650	**0.791**	0.746	0.769	**0.832**	0.819

**Table 2:** Performance comparison using SVM, SSL, and DNN with training set using 3 months of data.

Validation set period	Accuracy			ROC			F1 Score		
	SVM	SSL	DNN	SVM	SSL	DNN	SVM	SSL	DNN
2019.07.01-2019.09.30	0.748	**0.829**	0.809	0.657	**0.792**	0.746	0.760	**0.796**	**0.796**
2019.08.01-2019.10.31	0.742	**0.836**	0.813	0.660	**0.806**	0.756	0.761	**0.803**	0.800
2019.09.01-2019.11.30	0.746	**0.834**	0.816	0.642	**0.813**	0.765	0.765	0.801	**0.806**
2019.10.01-2019.12.31	0.757	**0.838**	0.814	0.658	**0.811**	0.759	0.775	**0.808**	0.802
Average	0.748	**0.834**	0.813	0.654	**0.805**	0.756	0.765	**0.802**	0.801

**Figure 10:**
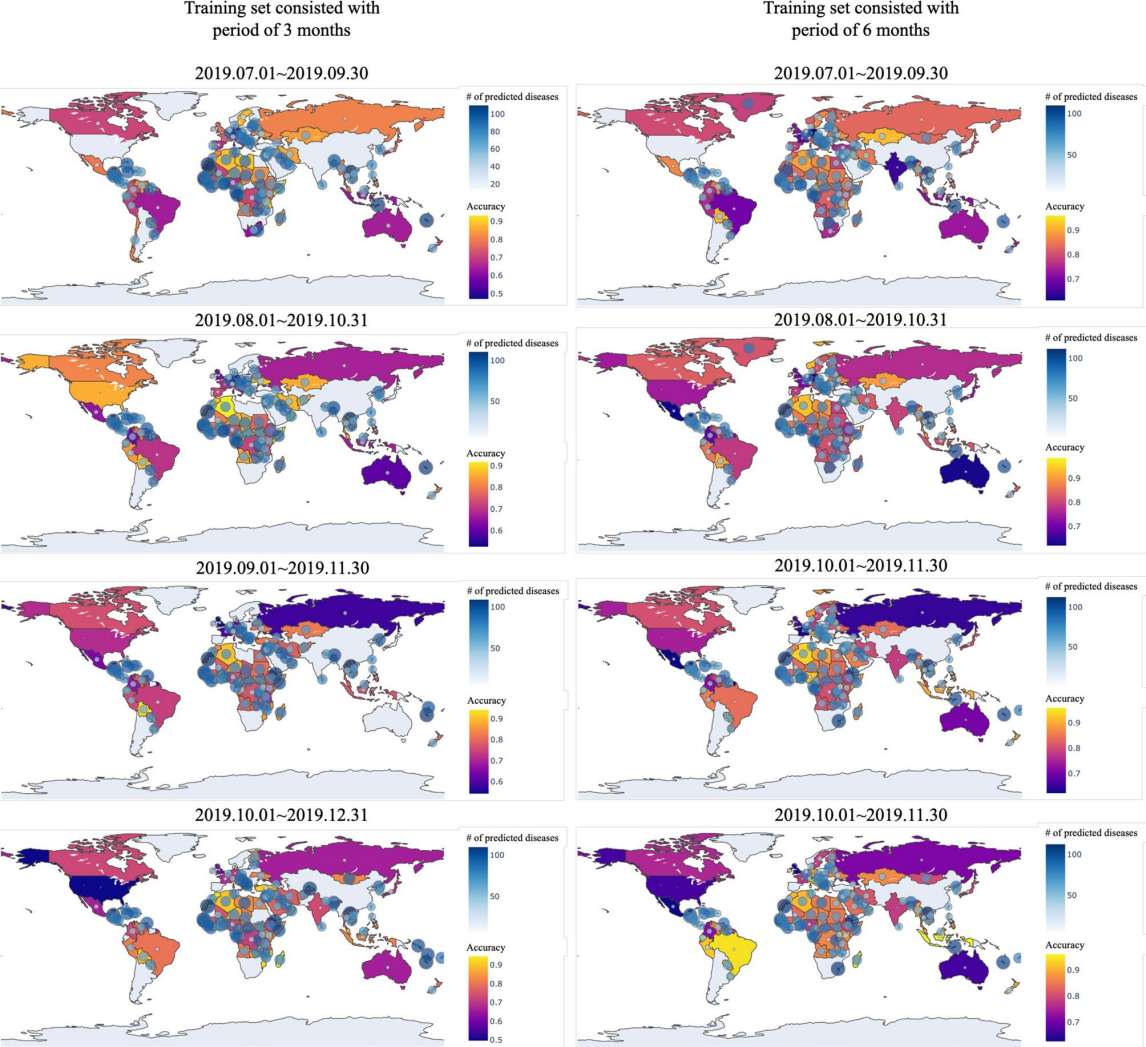
Prediction accuracy of SSL by each country: blue circles in the map indicate the number of predicted diseases for the country, and the closer the yellow, the more accurate the blue, the lower the accuracy. The figure was created in Python3 using the Basemap Toolkit.

In Fig. 10, the prediction accuracy of SSL for 8 different experiments are shown in the world map. Some countries are not colored in the map because every kinds of diseases were mentioned through media articles in these countries. In other words, these countries had no diseases to be predicted. While prediction accuracy of most countries is over 0.8, there are some countries showing very low prediction accuracy. This is because countries showing low accuracy contains only small number of diseases to be predicted. Thus, a wrong prediction of any one would significantly reduce the accuracy of the prediction.

## Discussion

In this research, the potential of utilizing media data to predict if an infectious disease will break out or not in a particular country using three of the most widely used machine learning models showed reasonable prediction performances. The occurrence of infectious diseases varies depending on many different reasons, such as climate, lifestyle of countries, diplomatic relations between countries, or population and so on. Therefore, similar types of infectious diseases are likely to occur in countries with similar comprehensive environments. In other words, countries with similar severity of various types of infectious diseases can be regarded as countries with similar environments. Thus, countries with similar infectious disease outbreak patterns can be identified by analyzing the patterns of severity of various types of infectious diseases between countries. Moreover, various existing studies have shown that the degree of incidence of media articles related to a specific infectious disease occurring in a specific country may indicate the severity of the disease in that country. Thus, this study attempted to predict the occurrence of specific infectious diseases in a specific country by analyzing the outbreak patterns of media articles related to various infectious diseases between countries. As the suggested method uses only media articles, even developing countries that have not yet constructed any disease surveillance systems, are able to forecast if particular infections will occur or not, because there are no critical limitations to accumulating such media articles.

Despite these advantages, further studies should be carried out in the near future to resolve several obstacles. First of all, the periods of training data and validation data were set by dividing the period of the year by the fourth quarter or half of the year from the data used for prediction, but a more systematic data time-setting strategy is needed, such as considering seasonal infectious diseases. Moreover, as Medisys does not provide old posts data, only about a year’s worth of data has been accumulated now since we started to collect Medisys data from the end of 2018. Therefore, when more data is collected, it is necessary to make predictions by later gathering additional data, and setting the duration of the training data to at least 1 year.

**Figure 11:**
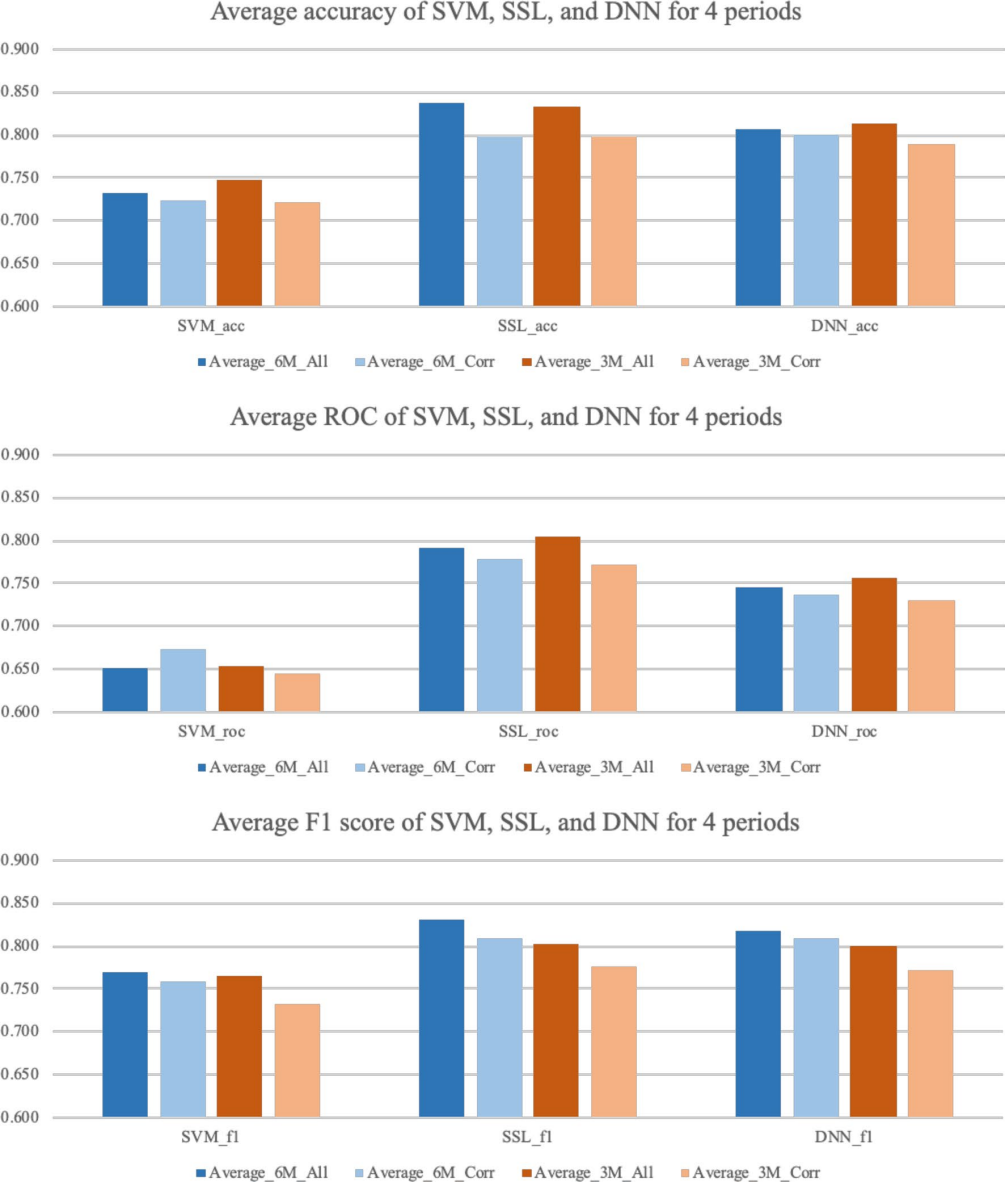
Prediction performance comparison between models using all countries and models using countries having a disease occurrence pattern correlation coefficient of over 0.6.

Second, even though all three models showed reasonable performance, it is necessary to discover methods to improve the performance of the prediction models. In this study, we also looked at whether it would be possible to improve the performance of prediction models when models are trained with data consisting of countries that show similar infectious disease occurrence patterns. Therefore, the prediction models for each country are trained using only data from countries with a correlation coefficient of 0.6 or higher, and Fig. 11 shows the performance of each prediction model. It was expected that the prediction results using data would consist of countries having disease outbreak pattern correlation coefficients of over 0.6; however, generally they showed worse performance. From this result, it can be inferred that even from countries where infectious disease patterns are dissimilar, the prediction model extracts useful information, and trains them. Thus, in further works, instead of feature selection, accumulating useful data, such as global air passenger data, which can represent the degree of relation between countries, is required to utilize them as weight for prediction models.

## Conclusion

The biggest reasons why it is not easy to predict the exact incidence of infectious disease is that a variety of characteristics, such as the nature of the infectious disease, the geographical characteristics of where the infectious diseases occur, the characteristics of people living in the country, the way people live, the kinds of things that spread infectious diseases, and the degree of exchanges between countries should all be taken into account. Furthermore, as time goes by, the weather changes due to global warming, digitalization changes people’s lifestyles, and for many reasons, the status of countries that trade frequently with each other changes. For these reasons, it is challenging work to create a predictive model that takes all of these characteristics into account. However, as the pattern of infectious diseases varies from country to country due to these various reasons, infectious disease incidence data by country can be considered to contain this information. Therefore, in this research, we tried to predict which disease will occur or not in particular countries, analyzing media data accumulated from Medisys using several machine learning models. Our suggested method showed reasonable prediction performance by the three different trending machine learning models SVM, SSL, and DNN. It is thought that the proposed model could be used to prepare for the future outbreak of infectious diseases in various countries, including developing countries that lack proper disease surveillance systems.

## Data Availability

The datasets used during the current study are available from the corresponding author on reasonable request.

## Abbreviations

SVM: Support vector machine
SSL: Semi-supervised learning
DNN: Deep neural network
MERS: Middle east respiratory syndrome
SIR: Susceptible infected recovered
ROC: Receiver operating characteristic
